# To scrape or not to scrape, this is dilemma. The post-API scenario and implications on digital research

**DOI:** 10.3389/fsoc.2023.1145038

**Published:** 2023-03-16

**Authors:** Domenico Trezza

**Affiliations:** Department of Social Sciences, University of Naples Federico II, Naples, Italy

**Keywords:** digital data, scraping, post-API, social media, digital methods

## Abstract

**Introduction:**

This article aims to investigate the potential impact of restricted social data access on digital research practices. The 2018 Cambridge Analytica scandal exposed the exploitation of Facebook user data for speculative purposes and led to the end of the so-called “Data Golden Age,” characterized by free access to social media user data. As a result, many social platforms have limited or entirely banned data access. This policy shift, referred to as the “APIcalypse,” has revolutionized digital research methods.

**Methods:**

To address the impact of this policy shift on digital research, a non-probabilistic sample of Italian researchers was surveyed and the responses were analyzed. The survey was designed to explore how constraints on digital data access have altered research practices, whether we are truly in a post-API era with a radical change in data scraping strategies, and what shared and sustainable solutions can be identified for the post-API scenario.

**Results:**

The findings highlight how limits on social data access have not yet created a “post-Api” scenario as expected, but it is turning research practices upside down, positively and negatively. On the positive side, because researchers are experimenting with innovative forms of scraping. Negatively, because there could be a “mass migration” to the few platforms that freely grant their APIs, with critical consequences for the quality of research.

**Discussion:**

The closure of many social media APIs has not opened up a post-API world, but has worsened the conditions of making research, which is increasingly oriented to “easy-data” environments such as Twitter. This should prompt digital researchers to make a self-reflexive effort to diversify research platforms and especially to act ethically with user data. It would also be important for the scientific world and large platforms to enter into understandings for open and conscious sharing of data in the name of scientific progress.

## 1. Introduction

How much have digital research practices changed in light of the changing accessibility of platform data? This is the main question that has been guiding the work and which aims to clarify whether and how much indeed we can speak of a post-API scenario, as is now the practice in the literature (Freelon, 2018; Bruns, 2019). The background hypothesis is that the closure of most Application Programming Interfaces (APIs) or their severe restriction by many social web platforms has, in some way, changed the practices of digital researchers. It all seems to have started with the well-known Cambridge Analytica (CA) scandal, which brought to light the ambiguous buying and selling of personal data from Facebook, for profiling users for political purposes. This scandal lifted the lid on a very severe privacy issue in the digital world. The expected consequence was the stop of many social platforms on free access to their data (Freelon, 2018; Tromble, 2021; Özkula et al., 2022).

This has made the scenario of digital scholars much more complex, because empirical research, especially quantitative research, relies on the availability of data. The risk, which I consider in this contribution, is that the availability of data more or^1^ less easily affects so much the way digital research is done, resulting in some platforms being “over-studied,” others being neglected by research, regardless of their popularity. From this premise arise the three questions of the paper: how much have constraints on digital data access changed research practices? Can we speak of an effective post-API era, that is, has there been an effective migration to other data scraping techniques? Finally, what are the possible shared and sustainable solutions for the post-API scenario? In the first case, I intend to explore the possibility that the difficulties in accessing data from popular platforms such as Facebook has actually radically affected how data is collected. In the second case, the hypothesis is that the scientific use of open platforms such as Twitter exploded after 2018. In the third case, this change since 2018 is possible that it has produced the need for a new consciousness toward data processing, especially from an ethical perspective.

Here, for simplicity of language and to follow most of the literature in the field, I mean by post-API simply the phase following the CA scandal and the consequences on data access limitations. In addition, I keep the concepts of “APIs” and “Web data scraping” (not infrequently used as synonyms) quite distinct, as for the former concept refers to the official applications provided by platforms for “controlled” data downloading. The second, on the other hand, is a concept that also embraces either manual data retrieval practices or handcrafted devices for scraping data from the Web.

## 2. Digital data and methods for scraping

The spread of Web 2.0 and social media have accelerated the process of “datafication” of society (Van Dijck, 2014; Amaturo and Aragona, 2019). They have become the information centers of our consumption, habits, and status. Therefore, social media are suitable for studying human and social phenomena, both for scientific research and for less noble issues such as marketing (political, economic, etc.). Social research today is increasingly convinced that the study of society cannot neglect digital phenomena and the content of social platforms. On the other hand, it no longer makes sense to divide the “real” from the “virtual” (Rogers, 2009). In other words, “digital” reality represents the evolution of our society that produces new data with which to interact through digital methods (Rogers, 2013; Amaturo and Aragona, 2019).

The epistemological issues surrounding digital data are particularly relevant to understanding the implications of API restrictions post-2018. Scholars such as Kate Crawford, Rob Kitchin, and Törnberg & Törnberg have explored how the limitations on accessing and using digital data can have profound social and political implications. For example, Crawford has argued that the rise of algorithmic decision-making based on limited data can have negative consequences for marginalized groups (Crawford and Joler, 2018). Kitchin has also highlighted how the power to control and regulate digital data is not evenly distributed and can reinforce existing power structures (Kitchin, 2014). Törnberg and Törnberg (2018) highlight the need to move beyond a technologically deterministic view of digital data and instead consider the social, political, and economic contexts of data production and use. Similarly, Evelyn Ruppert's work on the politics of data highlights how restrictions on data access and use can have far-reaching consequences for both researchers and the broader public (Ruppert, 2019). Understanding these complex issues is crucial for researchers seeking to conduct digital research post-2018 and navigate the limitations on API access and use.

It is a paradox, but this wide availability does not result in easy access to data. Restrictive privacy policies and the very nature of digital data (volatility, volume, size) make it difficult to obtain. Techniques and software for building digital databases are now highly desired. Two major families of data collection in the World Wide Web can be recognized. The first refers to digitized or virtual methods (Rogers, 2009), that is, social research techniques adapted to the online environment. For example, web surveys or long-distance interviews implemented with online services. On the other hand, the second includes digital methods, with Web data scraping (WDS) and techniques involving the use of Application Programming Interfaces (APIs). In general, WDS can be defined as the process of extracting and combining content of interest from the Web in a systematic way. WDS helps to take raw data in the form of HTML code from sites and convert it into a usable structured format. Not only that, manual extraction by simple copy-and-paste from Web pages is also part of WDS activities. Because a huge amount of heterogeneous data is constantly being generated on the Web, WDS is widely recognized as an efficient and powerful technique for collecting big data (Bar-Ilan, 2001; Mooney et al., 2015). To accommodate a variety of scenarios, current WDS techniques have become customized, moving from small *ad-hoc*, human-assisted procedures to the use of fully automated systems capable of converting entire Web sites into well-organized datasets.

WDS predates API extraction by more than a decade, but it has often been marginalized by APIs that have become easier to access. It is more flexible than API extraction because it can be used on most Web pages, not just those that offer APIs. Thus, it is a more versatile technique and, more importantly, more durable than API-based techniques, which, as will be seen below, are highly subject to the restrictive policies of platforms. Data scraping involves three main requirements that researchers must address. First, the scraping of social media data is a way to collect information from human subjects, so it must meet ethical standards accepted by the scientific community, such as preserving user privacy (Markham and Buchanan, 2012). Second, connected with the first point, a data scraping procedure should comply with the terms of service (TOS) of the platform from which the data is collected (Mancosu and Vegetti, 2020). Third, the data collected must comply with legal regulations that protect people's data. In particular, as of May 2018, the European Union (EU) has issued a new regulation for individual researchers and companies, the General Data Protection Regulation (GDPR), one of the most stringent data protection laws currently in place, which provides a set of restrictions to which scientific researchers using human subjects must adapt. And which, as will be seen below, is revolutionizing the way we approach data.

## 3. APIcalypse now?

For many years, the big corporate of the Web, such as Google and Facebook, have been exclusive owners of much of our data, handing it over to third-party companies with ambiguous goals. Up to that time, research made extensive use of this data, sometimes even with little care about privacy rules. The uncontrolled downloading of data was allowed mainly through filtering devices with platforms, called APIs, by which access keys were provided to the analyst to initiate scraping operations. APIs were the main tool by which researchers collected behavioral and digital trace data from Facebook (Fiesler and Proferes, 2018).

The scandal of CA, the company that has been using Facebook users' data for speculative purposes, has burst the bubble of precarious Internet privacy in 2018 (Hinds et al., 2020). Many web platforms have decided to beat a retreat on free data sharing. Shutting down the huge free flow of user data on major platforms has potentially reduced the prospects for studying an important slice of human society.

Among the likely consequences, discussed in the literature, at least four of them deserve to be highlighted. The first is that research would have flattened only on easily accessible data, thus neglecting varied and mixed approaches on different online contexts, at the expense of exclusively API friendly environments (Bruns, 2019; Caliandro, 2021). The second is that analyses are often conducted on secondary datasets, sourced on web depositories and thus often constructed with other goals in mind (Perriam et al., 2020). Finally, frequent violation of TOS has been reported, a consequence of researchers' attitude of circumventing social platform policies to retrieve data, such as through the use of unclear scraping techniques (Bruns, 2019). Also not insignificant is the possible contraction of data-based approaches, at the expense of less empirical approaches that bypass the burden of constructing digital data (Puschmann, 2019).

Researchers who have asked this kind of question while exploring the characteristics of the post-API scenario come to almost the same conclusion: the difficulty of finding data after 2018 has radically altered digital research, and the consequences, still in its infancy, could be traumatic and lead to heavy biases for present and future digital research outcomes (Freelon, 2018; Bruns, 2019; Caliandro, 2021).

Not everyone agrees with this negative hypothesis. Some data scientists and scholars of digital environments believe that this “apocalyptic” revolution has not occurred. On the contrary, the restrictions on platform data access probably can help improve the ethical and legal scenario on digital scraping (Fiesler and Proferes, 2018; Caliandro, 2021; Tromble, 2021). On the other hand, having unlimited data would have turned researchers away from their privacy obligations to users, and encouraged ambiguous data collection practices in violation of social media rules (Tromble, 2021).

Given the changes in research practices since 2018, it remains unclear to what extent these changes have affected different stages of research (such as data construction and analysis). Prior to API restrictions, independent researchers had easy access to public user profile information, comments, and reactions to public posts *via* third-party APIs. This facilitated studies on the societal impact of social media. However, the discontinuation of these APIs has effectively prevented independent researchers from conducting observational studies on topics such as political and social behavior, as well as the spread of real and fake news, news network structure, and political engagement dynamics on Facebook's platform. As Facebook's APIs were the only means by which third parties were authorized to collect data from the platform, these restrictions have had a significant impact on research possibilities.

**Figure 1:**
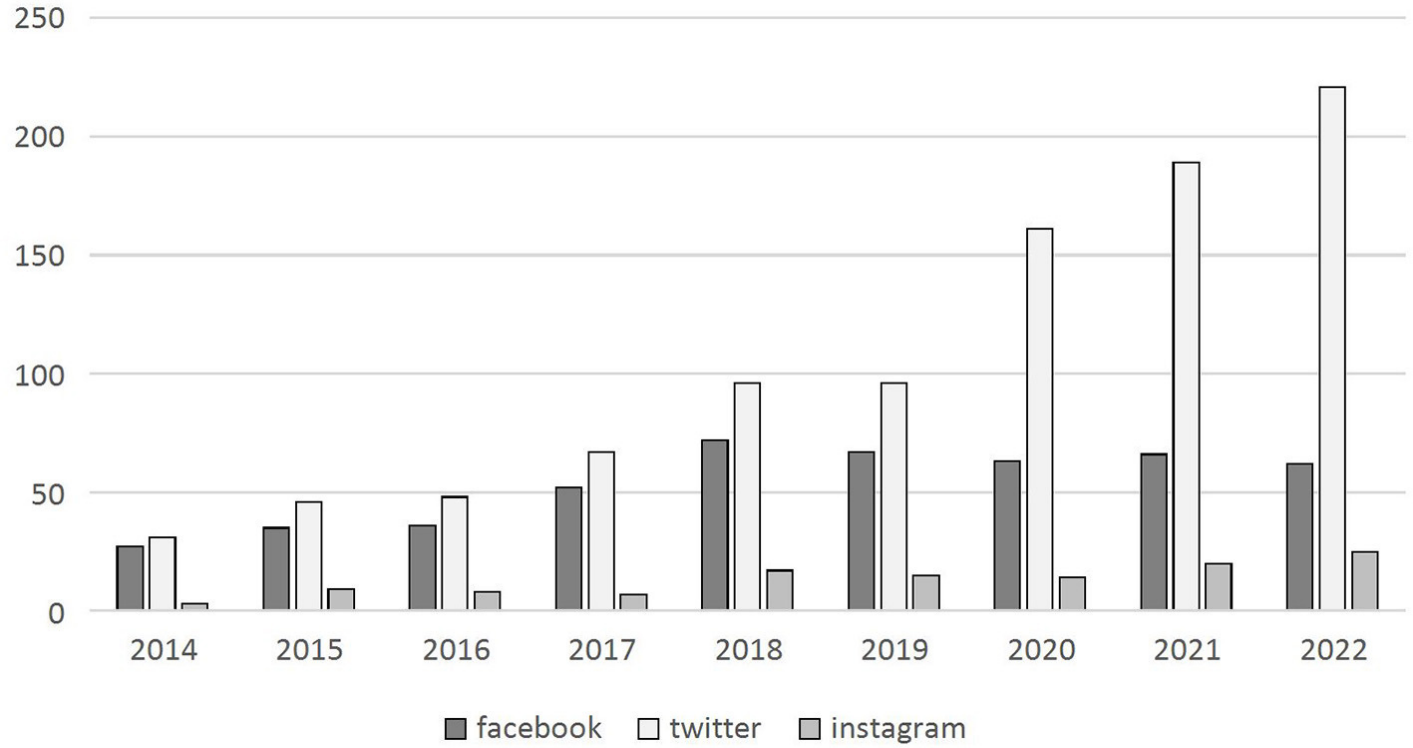
Publications on data scraping (2014–2022). Elaboration on dimensions data.

This has raised general concern among scholars, and sparked a debate around (potential) alternative ways to access crucial data to pursue social research on Facebook (Freelon, 2018; Bruns, 2019; Venturini and Rogers, 2019). Meanwhile, if one looks at the scholarly output built on digital data one realizes that progressively this is shifting toward Twitter, with a significant jump since 2018, the year of Facebook's closure (Figure 1). The paradox, however, is that information has not disappeared from the web, but instead is still publicly available. Scholars have also begun to discuss possible alternative methods of obtaining Facebook data, in what some scholars have already called the “post-API era” (Freelon, 2018; Bruns, 2019). Moreover, it appears that other platforms will follow suit by making it more difficult, if not impossible, to collect behavioral data easily and securely.

## 4. Materials and methods

### 4.1. Study and research questions

The study was created to explore the post-API scenario presented in the literature and to give, even if partially, an answer to these three questions:

How have constraints on digital data access changed research practices?

Can we speak of an effective post-API era, that is, has there been an effective migration to other data scraping techniques?

What are the possible shared and sustainable solutions for the post-API scenario?

To achieve this, we formulated three open-ended questions that asked participants to reflect on their experiences with digital research methods. The first question asked participants to identify any challenges they faced during the research process, while the second question focused on the most commonly adopted strategy for collecting data online. The third question sought to determine participants' preferred social platform for data collection.

The use of open-ended questions in research is a common approach and is advocated by several researchers, including Patton (2002) and Creswell (2014). Patton emphasizes the importance of open-ended questions in qualitative research, as they allow participants to express their experiences and views in their own words, leading to a more complete understanding of the phenomenon under investigation. Similarly, Creswell notes that open-ended questions are useful in exploratory research, as they allow the researcher to uncover a range of perspectives and experiences that may not be captured with closed-ended questions.

This method, which used asynchronous questions, encouraged the selected researchers to engage in an exercise of self-reflexivity on their digital research practices. This approach allowed the researchers to explore issues in-depth that are rarely reported in scholarly communications on digital research and are only mentioned in contributions to digital epistemology.

To provide more information on the methodology used to frame and analyze the responses, we can draw on the approach of Braun and Clarke (2006), which is a widely used framework for thematic analysis. This method involves several steps, including familiarizing oneself with the data, generating initial codes, searching for themes, reviewing and defining themes, and finally, producing the report.

### 4.2. Study group

The selection of researchers to be interviewed was done in the following way: the query search of the Scopus database was set up using certain keywords such as “digital methods,” “digital data,” “web analysis,” and “social media communication.” The top 18 Italian researchers by number of publications were contacted, considering the following quotas: 6 researchers for each of the three prevailing methodological approaches (mixed, qualitative and quantitative)^2^. Within these quotas, a precise selection criterion was not to include researchers from the same department. An email was sent to the identified researchers, with a brief motivation and with the survey form attached, customized for each researcher, with a brief presentation of my cognitive purposes, guaranteeing the confidentiality of the responses. The 18 researchers contacted all gave their willingness and returned the completed file within a maximum of 1 month.

## 5. Results

### 5.1. The three dimensions emerging from the responses

What are the three main macro-themes that emerge from the analysis of the responses regarding digital research? What are some of the challenges associated with collecting and interpreting data in digital research, and what are some of the strategies used to construct digital empirical material?

The three main macro-themes that emerge from the analysis of the responses regarding digital research are:

The complexity of doing research in digital environments, which involves dealing with different epistemological and methodological canons than traditional sociological research, and the challenge of constructing data due to the volatility and mutability of big data.

The use of WDS as an alternative method to APIs, which are secure but more constrained, and the impact on the platforms studied, such as the preference for social Twitter as an “easy data” environment for analysis of social phenomena on the web.

The use of web-scraping as a “necessary evil” for constructing digital empirical material, which can sometimes circumvent the terms and conditions imposed by the platform and raise ethical issues related to data privacy.

Some of the challenges associated with collecting and interpreting data in digital research include the accessibility of data due to API-related difficulties, the lack of cognitive access to the logic that governs algorithmic processes, and the risk of “banalization” in interpretation due to naive overenthusiasm toward the potential of digital methods. Some of the strategies used to construct digital empirical material include the use of digital tools found on the net and mostly free, learning mining scripts through programming software such as Python or R, and web-scraping techniques, which are sometimes seen as a necessary evil but raise ethical concerns related to data privacy.

### 5.2. The problematic steps of digital research: Collecting and interpreting data

The design of digital research shares much of the structure of traditional design. What changes is the way the different stages are processed, which are affected by the radically different nature of digital data. Typically, the formulation of research questions is different because they should consider new issues such as the digital divide, or the indeterminacy of the population studied, according to a post-demographic perspective (Rogers, 2009). In this very different scenario from traditional research, information gathering emerges as the most problematic because it is affected by the peculiarities of online environments.

The limitations, as revealed by multiple responses, mainly concern the accessibility of the data ≪due to the restrictions of APIs≫ (quantitative researcher 1). API-related difficulties are also epistemological: what are the processes that govern such applications? What impact do they have on the return of data and thus its fidelity? This invokes the general theme of data as a social construct and not as an object in its own right. It applies especially to algorithmic processes, which are seemingly “objective” but certainly lack neutrality because they respond to predetermined logics (Aragona, 2021).

The logic of how the API works is often unclear: for example, the Standard 1.1 version of the Twitter API states ≪(...) the Search API is not meant to be an exhaustive source of Tweets. Not all Tweets will be indexed or made available *via* the search interface≫ (Twitter, 2021). So not all Tweets are extracted, and what criteria excludes some of them? The data policy is unclear on this point. It is clear, then, how the issue of not having cognitive access to the logic that governs these processes excludes the possibility of having total control over the data collection phase. A condition, this, that cannot be ignored in the later stages of analysis and interpretation.

The issue of interpretation is precisely the other obstacle that insiders warn has a high risk of being oversimplified and losing its scientific rigor due to the ≪naive overenthusiasm toward the potential and gnoseological scope of digital methods≫ (qualitative researcher, 2). It is then pointed out that the real challenge is not ≪the possibility of retrieving a data or producing a result, the real challenge for researchers is to actually extract meaning, practical, critical and theoretical knowledge≫ (mixed researcher, 1).

This is the question, of absolute importance to the scientific community, of not falling victim to totally “data-driven” views but of not being impervious to perspectives that can play a decisive role in the interpretation of results.

All this needs an adequate problematization of the data, its limits and potential, its actual quality and its real cognitive power when theorizing on it is built with the intention of intervening on the reality under study to improve it or to allow a better understanding of it not only to insiders but also to gradually broader and broader audiences that can potentially be involved.

### 5.3. The construction of digital data. Web scraping as a “necessary evil”

What are the main strategies for constructing digital empirical material? Although some answers emphasize the relevance still of digitized methods *via* websurvey or digital ethnography, the difficulty of accessing digital data *via* APIs makes people devise a wide variety of Web-scraping strategies. Applications range from scripts in well-known programs (such as R and Python) to digital tools found on the net and which, depending on limitations or access, have enabled data retrieval on different sides (such as Crowdtangle, but also fanpagekarma and the similar). Again, digital tools available on the net and mostly free.

It is a fact that, indeed, the restrictions that started in 2018 have complicated scholars' research tasks. However, this has by no means encouraged throwing in the towel. While for some the solution has been to rely on digital tools (in some cases for a fee) hooked into social APIs, others, on the other hand, have had to rediscover skills as true data scientists, learning mining scripts through programming software such as Python or R.

Still, in other cases the need for do-it-yourself solutions based on WDS techniques emerged, however, well aware of the ethical sensitivity of certain issues. Web-scraping is, in fact, always a risky data collection option as it can sometimes “circumvent” the terms and conditions imposed by the platform. API querying, which ensures that data is used/accessed in a manner compliant with the terms and conditions and, according to some, always preferable.

It is equally true that web-scraping is sometimes a ≪necessary evil (...) as long as it is used under precise data collection and management conditions≫. Here is where the ethical issue of data privacy may become an important issue for researchers as well. Greater awareness on the topic certainly could have positive effects on the quality of doing research.

### 5.4. Twitter as the “easy data” platform

Among social media platforms, Twitter appears to be the most frequently studied by researchers. Simply because it is a platform from which data can be downloaded very easily, quickly and in large quantities. In addition, the data and metadata available and downloadable through Twitter's API is in a format that is very easy to handle in analysis, whether qualitative or quantitative.“

Twitter's popularity among experts is due to the easy availability of the API, as the researchers interviewed confirm. They, in fact, are careful to point out that Facebook has much fewer public data and has stricter limits on its application programming interface. On the other hand, Tufekci (2014) calls Twitter the “model organism” of big data, precisely because the platform's developers have implemented easy submissions to request APIs for tweets.

This “data gold rush” would lead researchers to use Twitter content (both tweets and profile information) to examine all kinds of aspects of human interaction. However, the non-randomness of the data acquired through these APIs means that Twitter studies have drawn conclusions based on substantially biased inferences. None of the public APIs guarantees the acquisition of all tweets matching the parameters of a query. Indeed, Twitter's developer documentation makes it clear that the search API will not return all tweets, and the streaming API limits captures when query parameters correspond to more than 1% of the total volume of tweets produced globally at any given time (Twitter, 2021).

## 6. Discussion

The reflections that arise from this study can only be partial and, above all, not generalizable because the number of researchers interviewed is very limited and confined to the Italian scene, which could report even significant differences with the foreign world. Despite this, this exploration has tried to give an answer, although not definitive, to three questions that I try to develop below.

How have limitations to digital data access changed research practices? It is difficult to contest the thesis that restrictions to APIs have complicated the researcher's work in Web contexts. According to the direct accounts of those who “get their hands dirty” with digital data, indeed there has been a setback to empirical documentation construction practices. It has been perceived as the twilight of the “Data Golden Age” (Bruns, 2019). On the other hand, when it comes to the difficulties of retrieving data on the Web, the paradox is obvious: on the one hand, the humanities and social sciences are constantly increasing epistemological and methodological reflection on the availability of huge amounts of data (Amaturo and Aragona, 2019); however, on the other hand, the actual tools to retrieve these data are becoming more and more residual.

Can we talk about an effective post-API era? Some platforms more than others have restricted access to their data. The case of Facebook is exemplary: under the spotlight for the CA scandal, it has decided to turn around its API release. The closure of the data “spigot” by the most popular social media has evidently created discouragement and dissent in the scientific community. The outcomes, however, did not include a mass migration to non-API forms of extraction; rather, there was a shift to other, more “easy data” platforms such as Twitter. As confirmed by some responses, favoring certain platforms over others is also a predictable symptom of easier access to its data. The risk is that all of this may direct digital research, reducing the quality of scientific production.

What are the possible solutions for the post-API scenario? Web platforms that release their data easily and especially for free are becoming the preferred contexts of study. On the other hand, this invokes the epistemological urgency of having full knowledge of the construction process and theoretical foundations underlying digital data (Amaturo and Aragona, 2019). There are two proposals that, between the lines, those who responded put forward. The first, in the shortage of applications to exhaustively collect digital data, research has sometimes been “encouraged” to bypass data privacy rules, often violating the platforms' terms of service. It is important, then, to create awareness of the ethical issues surrounding digital search, since it always involves data that refers to people. The second might be to create synergies between Academic Institutions and large Web platforms to promote the free sharing of data, at least at the scientific level. A few attempts have already been made in the past (e.g., Social Science One, founded by Facebook) but the results are not very appreciable, considering that the platform has the final say on research objects. This suggests that the transition to the post-API research world should be non-traumatic and should not completely disrupt the practices of digital researchers. As seen, the limited availability in accessing platform databases is creating a competition for those few environments that still leave their APIs open. A really important point, then, becomes the integration of scraping tools and platforms to preserve the “representativeness” of the research object. These mixed practices of data construction are gradually being consolidated.

The typology in Table 1 summarizes these concepts that have emerged from the content analysis of responses. On the one hand, there is the degree of researcher intervention (manual or automatic), on the other hand, there are the techniques (API or WDS). Four types of practices and related tools with predominantly adopted platforms emerge from the intersection. This typology suggests that each practice probably fits well with specific platforms. For example, those studying Twitter are more inclined to use programming scripts allowing the downloading of tweets, whereas, the known restrictions related to Facebook have probably pushed researchers of this social to migrate toward manual, copy, and paste type WDS strategies. As can be imagined, the methodological orientation of the researcher certainly plays a non-marginal role in the choice of data extraction strategies: while mixed researchers are comfortable among different modes of data retrieval (from API to copy and paste), quantitative researchers are more inclined to API forms of extraction (and thus more oriented to the Twitter platform), on the other hand, qualitative researchers are the ones who try experimenting with more varied forms of extraction (and also more specific Tools), to study emerging platforms such as Reddit or TikTok.

**Table 1:** Typology of data scraping.

	**API**	**WDS**
MANUAL	Script (*R, Python*) → Twitter	Copy and paste → Facebook
AUTOMATIC	Tools API (*Storysaver*) → Instagram	Tools WDS (*Crowdtangle*) → all platforms

Constraints on platform data accessibility has certainly changed the scenario of digital research and digital methods. It has been observed, however, that this change has not meant moving out of the API era; rather, the ways of doing digital research have been reconfigured. Apparently, this change is resulting in a fragmentation of the ways in which digital data is being constructed, which, depending on one's inclinations (qualitative, mixed or quantitative) and, most importantly, on the availability of time and money, is directed toward extraction strategies over others.

### 6.1. Conclusion

This article stems from a path of reflection that has accompanied and is accompanying me and my professorial colleagues' digital research work, especially in relation to the construction phase of empirical digital material. Although the Web is a gold mine of data, the data we need are not always immediately available to us and readily available. The Cambridge Analytica scandal of 2018 put a stop to the “gravy train” of digital data, especially for those on social platforms. Today, one has to undertake gyrations between applying for API access, subscribing to web scraping software, and good computing devices to create digital databases. These difficulties have not infrequently encouraged researchers to choose easier paths, perhaps reframing the questions and moving to analyse on the most “data-generous” platforms. Platforms such as Twitter, for example, still provide APIs with ease, putting doubts in the minds of proponents of the post-API paradigm. This, however, may have created quite a few biases: the most obvious one is that of the digital universe, only that small segment of users active on “easy-data” platforms would be studied. Therefore, it is not secondary for scholarly research to reason about the changes that take place in the big platforms' data policies, because these, as has been the case since 2018, can have very significant impacts on scholarly output and create relevant biases: do we study what is of interest or what is easy to study? API restrictions have had a major impact on academic research, and there is no doubt about that. However, rather than seeing it as a major loss, our research community can take advantage of this moment to critically reflect and improve. This initial exploration of the topic should stimulate digital research to reflect and rethink its scraping tools and the nature of digital data. These are pressing issues, because as heard directly from the voices of key research, there are indeed biases such as, for example, choosing one platform over another just because there is more accessibility.

Therefore, research should help explore new ways of scraping that are more ethical, that is, respectful of users' privacy; more sustainable, that is, open or low-cost; and, above all, more shared between the research community and the big platforms.

## Data availability statement

The raw data supporting the conclusions of this article will be made available by the authors, without undue reservation.

## Ethics statement

Ethical review and approval was not required for the study on human participants in accordance with the local legislation and institutional requirements. The patients/participants provided their written informed consent to participate in this study.

## Author contributions

The author confirms being the sole contributor of this work and has approved it for publication.

## Footnotes

^1^API stands for Application Programming Interface, which acts as an intermediary, allowing websites and software to communicate and exchange data and information.

^2^The methodological placement of each scholar was decided by comparing myself with some more experienced colleagues in my department and, of course, by considering their scientific output.
